# Sericin Protein: Structure, Properties, and Applications

**DOI:** 10.3390/jfb15110322

**Published:** 2024-10-29

**Authors:** Rony Aad, Ivana Dragojlov, Simone Vesentini

**Affiliations:** Department of Electronics, Information, and Bioengineering, Politecnico di Milano, 20133 Milan, Italy; rony.aad@polimi.it (R.A.); ivana.dragojlov@polimi.it (I.D.)

**Keywords:** sericin, silk, biobased materials, extraction processes, silkworm *Bombyx mori*

## Abstract

Silk sericin, the glue protein binding fibroin fibers together, is present in the *Bombyx mori* silkworms’ cocoons. In recent years, sericin has gained attention for its wide range of properties and possible opportunities for various applications, as evidenced by the meta-analysis conducted in this review. Sericin extraction methods have evolved over the years to become more efficient and environmentally friendly, preserving its structure. Due to its biocompatibility, biodegradability, anti-inflammatory, antibacterial, antioxidant, UV-protective, anti-tyrosinase, anti-aging, and anti-cancer properties, sericin is increasingly used in biomedical fields like drug delivery, tissue engineering, and serum-free cell culture media. Beyond healthcare, sericin shows promise in industries such as textiles, cosmetics, and food packaging. This review aims to highlight recent advancements in sericin extraction, research, and applications, while also summarizing key findings from earlier studies.

## 1. Introduction

Silk produced by *Bombyx mori* silkworms is composed of two main proteins, namely fibroin and sericin. Silk fibroin (70–75% of the silk proteins) is a fibrous protein secreted by the silkworm in two strands coming from the right and left sides of the excretory duct. The two strands are held together by the sericin (25–30% of the silk proteins), a globular protein being secreted at the same time and acting as the “gum” coating the fibroin strands and allowing them to stick to each other, forming the final silk thread. After being secreted, sericin loses its rubbery texture and acts as a rigid adhesive, binding the cocoon fibers as it forms. After 3–4 days, the weaving period stops, and the final cocoon is a rigid shell that preserves the chrysalis until its transformation into butterfly. Reelers and silk processors prioritize fibroin within the fiber exclusively, and traditionally, sericin, viewed as a secondary product, is discarded along with degumming water. Furthermore, unlike fibroin, which has enjoyed extensive use in non-textile sectors, particularly in biomedical applications, sericin was once considered cytotoxic and inappropriate for medical purposes [1]. Just as an example of this initial consideration, an extract from a highly cited article published in 2003 is reported. In the section regarding the utility of silk-based proteins in biomedical applications, it is stated “… it is clear that the sericin glue-like proteins are the major cause of adverse problems with biocompatibility and hypersensitivity to silk … if sericin is removed, the biological responses to the core fibroin fibers appear to be comparable to most other commonly used biomaterials…” [1]. This is just one example, but throughout the same article, there are many similar statements. Even though the same principal investigator published another paper in 2007 refuting the adverse effects of sericin, the original idea persisted, and the scientific community remained convinced that sericin should be removed and disregarded [1]. Nonetheless, recent breakthroughs in understanding sericin’s structure, properties, biocompatibility, and processability have highlighted its distinctiveness and the potential for its widespread adoption across various industries such as cosmetics, medical, food, fiber, and beyond [2]. Starting from the meta-analysis of the two main proteins with a particular focus on sericin, the attention will move on to the main molecular features that are responsible for the behavior of sericin. A detailed description of possible methods to extract sericin is also given with attention to methods that prevent protein degradation. Finally, consolidated applications and possible future directions and markets for the sericin are presented.

## 2. Meta-Analysis

### 2.1. Sericin-Based Studies

Various studies have been published from 1926 until 2024 on sericin, concerning various extraction methods of sericin, its inherent characteristics, and its vast range of applications. The literature abounds with comprehensive examinations of sericin’s multifaceted properties. In this dedicated section, a comprehensive meta-analysis study was systematically conducted scrutinizing sericin-related content across three prominent online databases: PubMed https://pubmed.ncbi.nlm.nih.gov/?term=sericin&sort=pubdate, (accessed on 10 June 2024), Science Direct https://www.sciencedirect.com/search?qs=sericin (accessed on 10 June 2024)), and SpringerLink (https://link.springer.com/search?query=sericin (accessed on 10 June 2024). As detailed in Table 1 below, the collective body of work surpasses 11,000 studies, predominantly comprised of research articles.

**Figure 1:**
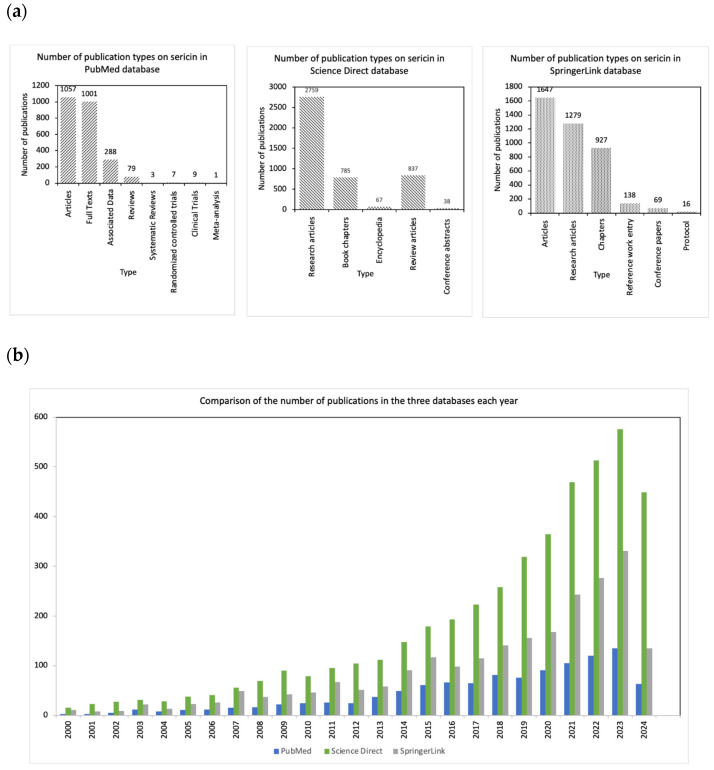
(**a**) Number of publication types on sericin in the three databases. (**b**) Comparison of the number of publications in the three databases each year.

The presented results stem from a comprehensive search across three prominent databases: PubMed, ScienceDirect, and SpringerLink. The graphical representation of the data is illustrated in Figure 1. Over the past decade, there has been a steady increase in the volume of sericin-related publications, with notable peaks in research output during specific years, possibly driven by advances in biotechnology and material science. Research articles constitute the predominant type of publication across all three databases, suggesting a high level of experimental engagement with sericin. This is followed by full texts and associated data in PubMed, review articles and book chapters in ScienceDirect, and chapters and books in SpringerLink. Notably, the prevalence of research articles highlights the continuous expansion of empirical studies, while the presence of review articles, book chapters, and monographs indicates a growing interest in consolidating and synthesizing knowledge about silk sericin. PubMed, with its focus on biomedical research, shows a stronger representation of studies on sericin’s medical applications, while ScienceDirect and SpringerLink cover a broader spectrum of industrial uses, including food packaging, textiles, and cosmetics. Furthermore, preliminary citation analysis suggests that research articles focusing on biomedical applications, particularly in drug delivery and tissue engineering, are among the most frequently cited in the literature, indicating their significant scientific impact and practical relevance. This analysis not only underscores robust engagement with sericin-related studies but also reveals the multidisciplinary nature of this research. However, gaps remain, particularly in exploring the full industrial potential of sericin in non-medical sectors, signaling opportunities for future investigation.

### 2.2. Fibroin-Based Studies

**Figure 2:**
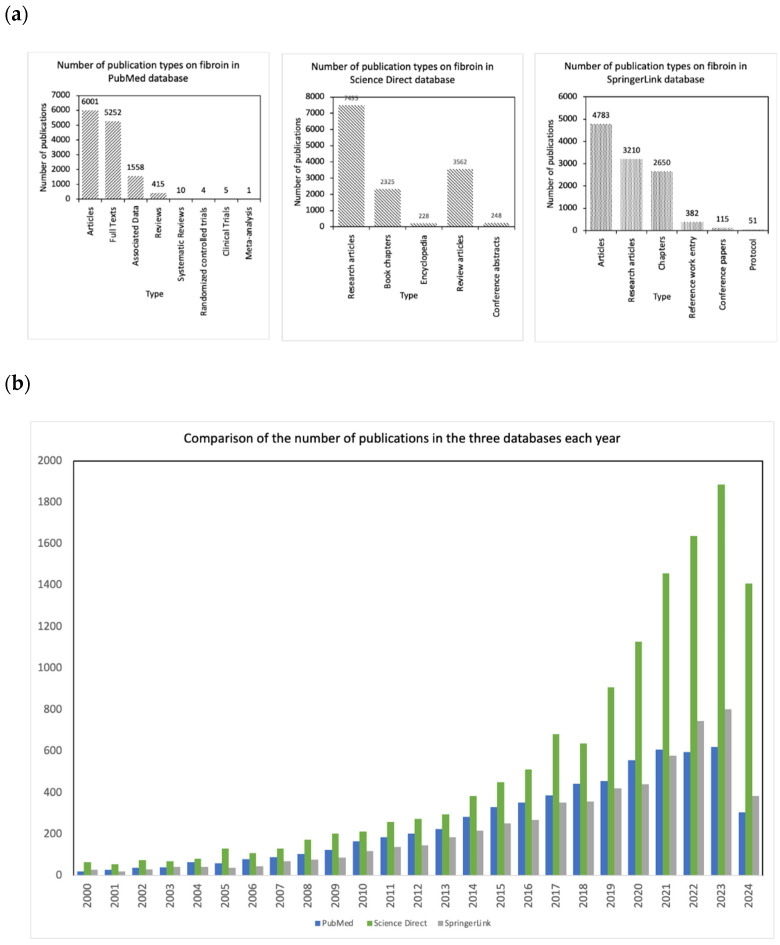
(**a**) Number of publication types on fibroin in the three databases. (**b**) Comparison of the number of publications in the three databases each year.

The findings presented in this meta-analysis are based on an extensive review of the literature concerning fibroin. An analysis of data from prominent databases such as PubMed, ScienceDirect, and SpringerLink reveals a steady increase in the volume of fibroin-related publications over the past decade as shown in Figure 2. This trend highlights a growing scientific interest, particularly in the fields of biomaterials, tissue engineering, and drug delivery systems. Research articles represent the majority of publications across these databases, indicating a significant level of experimental engagement with fibroin. Notably, the diversity of publication types, including review articles, book chapters, and technical reports, reflects the interdisciplinary nature of fibroin research, which spans various domains such as materials science, biomedical engineering, and pharmacology. PubMed showcases a stronger focus on the biomedical applications of fibroin, such as its biocompatibility and potential for drug delivery, while ScienceDirect and SpringerLink encompass a broader range of industrial applications, including textiles and environmental sustainability. Furthermore, preliminary citation analysis suggests that key articles focusing on the biomedical applications of fibroin are frequently cited, underscoring their significant scientific impact and relevance. This analysis not only highlights the robust engagement with fibroin-related studies, similar to those conducted on sericin, but also reveals emerging trends and opportunities for future research, particularly in exploring fibroin’s full potential in novel applications and addressing existing knowledge gaps in non-medical sectors. In conclusion, fibroin-related studies have historically dominated the landscape of silk-related publications, often overshadowing discussions surrounding silk sericin. However, in recent years, there has been a noticeable shift in focus toward sericin, an underutilized protein, highlighting its growing importance and potential applications. As evidenced by Table 2, the number of studies on silk-related topics exceeds 38,000, further confirming the disparity in attention between fibroin and sericin. This growing interest in sericin indicates an opportunity for future research to explore its unique properties and applications alongside fibroin, paving the way for a more balanced understanding of both proteins in the field of biomaterials.

## 3. Silk Sericin Structure and Properties

### 3.1. Chemical Composition and Structure

**Figure 3:**
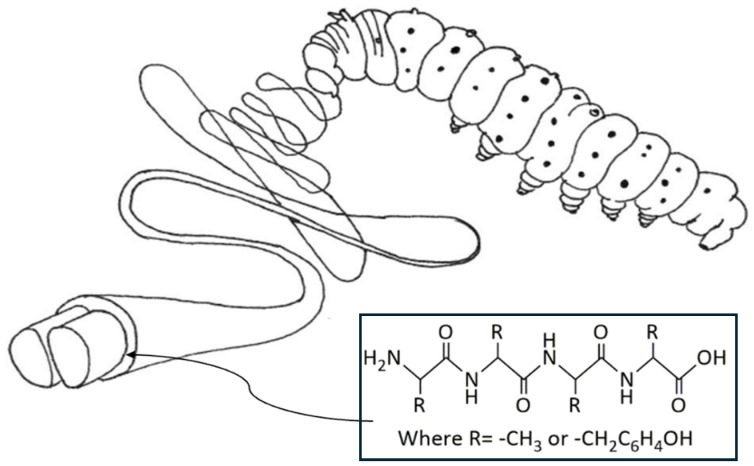
Chemical structure of sericin surrounding fibroin fibers from *Bombyx mori* silkworm.

Sericin, a protein extracted from silk, is known to be a “gluelike” protein since it holds the two fibroin filaments together, as shown in Figure 3 [2]. It is a globular protein; it consists of a random coil and β-sheets. Several factors affect the transition of sericin from random-coil structure to β-sheets. It is an easily occurring phenomenon in response to temperature, humidity, and mechanical properties. Sericin is soluble in water at temperatures from 50 °C and above [3]. However, at lower temperatures, sericin’s solubility decreases gradually; hence, the conversion of the random coil into β-sheets occurs, leading to the formation of a gel [2]. Kweon et al. studied the effect of poloxamer, a polymeric surfactant, on the gelation of sericin. The results showed that the addition of the poloxamer accelerated the change in sericin’s conformation from random coil to β-sheets. Also, it showed that the gelation of sericin runs faster with higher concentrations of sericin and higher temperatures [3]. Takasu et al., in chromatographic analysis of sericin, rationalized that the reason sericin in the silk gland is soluble in water, and that after being spun and dried it is not soluble, is that sericin molecules form β-sheets during the drying process, which renders them less soluble than the amorphous form [4].

**Figure 4:**
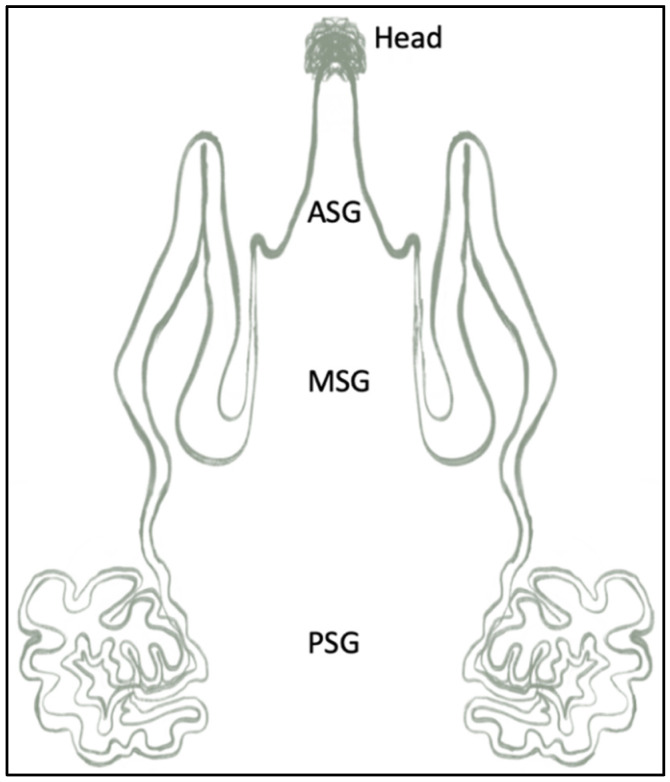
Representative sketch of the silk gland of *Bombyx mori* silkworm.

Sericin is a hydrophilic protein characterized by a high content of hydroxyl groups, carboxyl groups, and polar amino acids. While serine governs the sericin amino acid content, 17 other amino acids are present. Those are alanine, arginine, aspartic acid, cysteine, glutamic acid, glycine, histidine, isoleucine, leucine, lysine, methionine, phenylalanine, proline, threonine, tryptophan, tyrosine, and valine [5]. This intricate amino acid makeup plays a pivotal role in defining sericin’s variants, namely SER-1, SER-2, and SER-3, each characterized by unique amino acid contents and variable molecular weights [5]. The synthesis of sericin unfolds within the silk gland of *Bombyx mori*, a process intricately linked to its molecular composition. Considering the morphological and functional differences, the silk gland is divided into three parts, as shown in Figure 4, namely the anterior silk gland (ASG), which constitutes the excretory duct; the middle silk gland (MSG), which secretes three types of sericin; and the posterior silk gland (PSG) that secretes fibroin [2]. At the top end of the gland, there is a head, known as the spinneret, which is the fiber-spinning entity.

**Figure 5:**
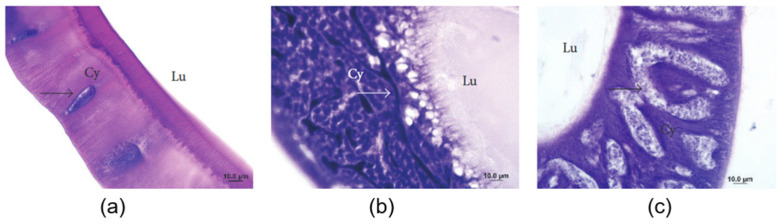
Photomicrographs of different regions (**a**) anterior, (**b**) middle, and (**c**) posterior, stained with hematoxylin and eosin. Cytoplasm (Cy), nucleus (arrows), and lumen (Lu). Adapted from [Kunz, R.I.; Brancalhão, R.M.C.; Ribeiro, L.D.F.C.; Natali, M.R.M. Silkworm Sericin: Properties and Biomedical Applications. *BioMed Res. Int.* **2016**, *2016*, 1–19]. Available online: https://doi.org/10.1155/2016/8175701 (accessed on 17 October 2024) [2].

The middle gland was subdivided into four distinct parts according to the density and morphology of the sericin synthesized and secreted [2]. Figure 5 provides photomicrographs of these distinct regions, highlighting the cytoplasm (Cy), nuclei (indicated by arrows), and lumen (Lu), thereby illustrating the structural characteristics essential for understanding silk production.

The amino acid composition of the sericin protein has been extensively reported in the literature. In Table 3 below, six studies were reported in chronological order showing the different sericin amino acid composition analyses over the years. In addition, in the last column, an average of the percentage values was reported for an overall evaluation purpose.

**Table 3:** Amino acid content of silk sericin for various references and their respective averages.

Amino-Acid	2000 [6]	2006 [7]	2009 [8]	2014 [9]	2015 [10]	2018 [11]	Average
Ala	4.60	4.30	3.86	4.30	ND *	3.28	4.07
Arg	2.80	4.90	6.16	3.60	11.95	4.71	5.69
Asp	19.10	18.80	17.64	14.80	14.00	11.52	15.98
Cyst	<0.05	0.30	ND *	0.10	ND *	0.03	0.14
Glu	4.10	7.20	7.31	3.40	3.30	2.91	4.70
Gly	12.20	10.70	9.89	14.70	23.20	12.60	13.88
His	0.90	1.70	1.81	1.20	1.13	2.05	1.47
Ile	1.40	1.30	1.04	0.70	0.91	0.34	0.95
Leu	0.60	1.70	1.44	1.40	2.08	1.05	1.38
Lys	10.20	2.10	3.05	2.40	3.18	2.33	3.88
Met	<0.05	0.50	0.11	ND *	0.77	0.13	0.38
Phe	0.40	1.60	1.08	0.30	1.29	0.53	0.87
Pro	0.80	1.20	0.59	0.70	ND	0.59	0.78
Ser	30.40	27.30	32.74	37.3	21.56	40.51	31.64
Thr	6.00	7.50	5.51	8.70	7.04	8.45	7.20
Trp	ND *	0.40	ND *	ND *	ND *	ND *	0.40
Tyr	3.80	4.60	4.63	2.60	6.23	5.42	4.55
Val	2.60	3.80	3.14	3.60	3.36	3.56	3.34

Values are shown in Mol (%).* ND: not determined.

### 3.2. Physical Properties and Characteristics

Sericin can be classified according to two different parameters: solubility and molecular weight.

#### 3.2.1. Solubility

Previously, sericin was classified into three different fractions, namely Sericin A, Sericin B, and Sericin C [12]. The examination of the amino acid composition of sericin was performed using paper partition chromatography. Sericin A, the one present in the outermost layer of the cocoon, is soluble in warm water, consists of almost 17.2% nitrogen, and the majority of its amino acids are serine, threonine, glycine, and aspartic acid. Sericin B is the one present in the intermediate layer; it consists of 16.8% nitrogen and has the same amino acids as Sericin A, in addition to tryptophan. Sericin C, which is the one found in the innermost layer, is insoluble in hot water and has a lower nitrogen content, 16.6%, compared to Sericin A and B. This fraction consists of the same amino acid content as A and B, in addition to proline. It should be noted that Sericin C can be removed from fibroin by treating it with a hot solution of an acid or an alkali. Sericin degrades easily due to its sensitivity to changes in pH and temperature, and its ability to dissolve in various solvents. Maintaining sericin’s structure is a challenge, when it comes to utilizing it in the synthesis of sericin-based materials. The reason lies behind the conventional degumming procedures which utilize elevated temperatures/pressures, and/or alkaline, which lead to sericin degradation [5].

#### 3.2.2. Molecular Weight

Silk sericin exhibits a spectrum of molecular weights influenced by various factors, with the primary determinant being its source. Whether extracted from natural sources, such as *Bombyx mori* cocoons, or synthesized through biotechnological techniques like recombinant DNA technology [5], these distinct methods contribute to variations in sericin’s molecular weight. Additionally, the specific extraction techniques used for isolating sericin from natural sources play a crucial role in determining its molecular weight. The molecular weight is affected by several variables, including extraction methods, temperature, pH, and processing time. This combination of factors results in a diverse molecular weight range for extracted sericin, spanning from 10 to 400 kDa [13]. Numerous extraction methods from silk cocoons contribute to this variability, encompassing high-temperature processes with [14] and without [15] high pressure, utilization of urea [14], acids [14], alkalis [14], enzymes [15], and microwave-assisted extraction [16]. With elevated temperatures and acid, extractions result in sericin with molecular weights of 35–150 kDa, and the alkaline extraction leads to molecular weights of 15–75 kDa. As Bascou et al. previously reported, water breaks down the peptide bonds of sericin, leading to its hydrolysis. Similarly in alkaline environments, the presence of alkalis further reduces its molecular weight. Additionally, elevated temperatures and high pressure accelerate the hydrolysis of the sericin macromolecule into smaller peptides. Furthermore, microwave heating penetrates deeper into the matrix and leads to greater hydrolysis of sericin, while steam heating methods result in the preservation of higher molecular weight fractions [16]. However, sericin extracted using urea is the only method that has shown different bands between 10 and >225 kDa using sodium–dodecyl sulfate–polyacrylamide gel electrophoresis analysis (SDS-PAGE). Nonetheless, the chemically produced sericin by the recombinant protein method has a molecular weight significantly lower than the one of the extracted sericin, which is almost 25 kDa [17]. Table 4 below outlines the impact of various common extraction techniques on the molecular weight of sericin. The section on extraction techniques provides detailed information about previously employed methods. As mentioned in a published study on the applications of sericin in biomaterials, it was conveyed that high molecular weight sericin exhibits functionalities in functional biomembranes, hydrogels, and functional fibers and fabrics. Nevertheless, the lower molecular weight sericin is applied in the cosmetic, health, and medical industries [18].

**Table 4:** The different sericin molecular weights according to the extraction/production method and their relative applications.

Method of Extraction/Production	Molecular Weight (kDa)	References
High temperature	100–200	[15]
High temperature–high pressure	25–150	[14]
Urea-based	10–225	[14]
Acid-based	50–150	[14]
Alkali-based	15–75	[14]
Enzymatic	5–25	[19]
Recombinant protein	≈25	[5]

### 3.3. Biophysical Characteristics

**Figure 6:**
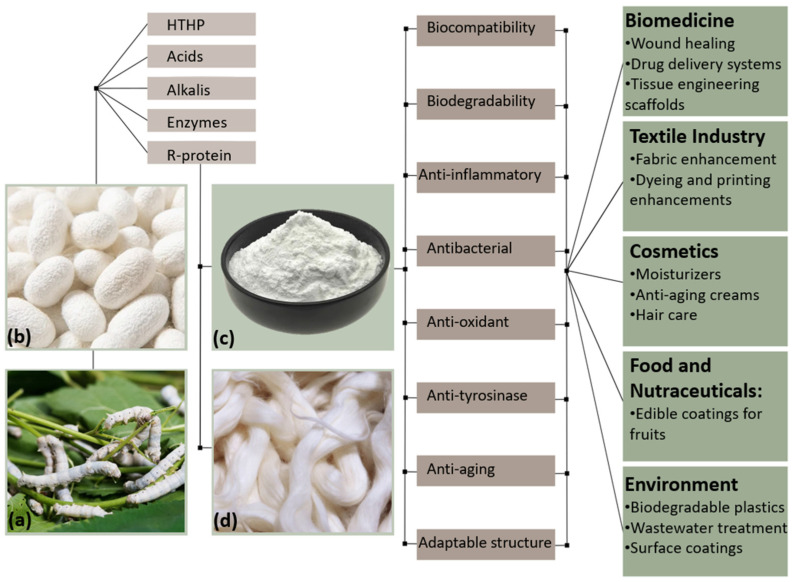
Sericin’s various characteristics and applications. (**a**) Silkworm *Bombyx mori*. feeding on mulberry leaves; (**b**) Cocoons produced by *Bombyx mori*. silkworms; (**c**) Sericin powder recovered from different extraction procedures; (**d**) Fibroin fibers. HTPT: high temperature, high pressure. R-protein: recombinant protein.

Sericin has emerged as a versatile biomaterial with diverse properties that enable its application across a wide range of fields. Biocompatibility is a key characteristic of materials intended for medical and biological use, as it refers to the ability of a material to interact with biological systems without triggering an adverse immune response [20]. Sericin’s exceptional biocompatibility ensures minimal immunological reactions when in contact with living tissues, thus reducing the risks of inflammation or rejection [21]. This makes sericin particularly suitable for biomedical applications such as wound healing, tissue engineering, and drug delivery systems [22]. In addition to its biocompatibility, sericin’s inherent biodegradability [23] aligns well with environmentally friendly practices. It can be naturally broken down by proteolytic enzymes in biological systems, such as proteases, which hydrolyze sericin’s peptide bonds into smaller peptides and amino acids [23]. However, the long-term effects of sericin in biological systems remain unknown. This is primarily because further studies are needed to understand the impact of the byproducts of sericin hydrolysis. These byproducts, along with their reactivity and potential interactions with bioactive compounds in the body, require thorough investigation to ensure the safety and efficacy of sericin in biological systems in the long term. Sericin also exhibits a range of functional properties beyond biocompatibility and biodegradability. Its anti-inflammatory, antibacterial, antioxidant, and UV-protective capabilities open up further applications in skin care, wound healing, food packaging, and textiles. Sericin’s anti-inflammatory activity is associated with its ability to modulate the release of inflammatory cytokines, such as interleukin 1 (IL-1) and tumor necrosis factor-alpha (TNF-α), which are key mediators of inflammation [22,24]. Additionally, sericin’s antibacterial efficacy is attributed to its cysteine content. Cysteine, through its sulfhydryl group, forms weak hydrogen bonds with oxygen or nitrogen, resulting in reactive compounds that interfere with the enzymatic and metabolic processes vital to microorganisms, thereby providing sericin with antibacterial properties [22,25]. Its antioxidant activity is linked to its ability to scavenge reactive oxygen species (ROS), with high levels of serine and threonine enabling effective chelation of transition metal ions like copper and iron [26]. These antioxidant properties help mitigate oxidative stress, a key factor in skin aging, thus contributing to sericin’s anti-aging effects [27]. Furthermore, the presence of arginine and alanine in sericin’s structure allows it to bind tyrosinase, giving it anti-tyrosinase activity and positioning sericin as an effective agent in skin-related applications [21]. Figure 6 illustrates the process of extracting sericin from *Bombyx mori* cocoons and its transformation into final products for use in various fields. The figure highlights the extraction techniques employed and the key properties that make sericin a valuable biomaterial.

## 4. Extraction Methods

### 4.1. Degumming Process Overview

The extraction method, known as degumming, entails the meticulous separation of the silk sericin layer, colloquially referred to as gum, from silk fibroin fibers. This intricate procedure involves the hydrolytic or enzymatic cleavage of sericin’s peptide bonds, subsequently facilitating its extraction from the silk fibroin matrix. Various methods, including chemical, thermal, and biological, have been investigated to achieve the successful extraction of sericin. After degumming, further purification methods can be applied to isolate pure sericin.

### 4.2. Conventional Extraction Methods

Degumming silk by boiling off in soap solutions, including the prevalent use of Marseille soap derived from olive oil, has been practiced for over two centuries. The soap’s hydrolysis produces alkali, which aids in the breakdown of sericin from the silk thread; subsequently, the sericin is dissolved in water through the emulsification action of the soap. Typically, a degumming time of 90–120 min at boiling temperature is sufficient, with Marseille soap preferred for its high degree of hydrolysis. Despite its effectiveness, the use of Marseille soap poses economic challenges [28], including potential water quality issues affecting silk quality and the need for high soap quantities, leading to environmental concerns [29]. To address these challenges, mixtures of soap and alkali have been explored, accelerating degumming while mitigating pollution concerns, albeit with susceptibility to hard water. Various alkalis, including sodium silicate and sodium carbonate, have been investigated for their effectiveness in enhancing the degumming process and maintaining pH levels conducive to efficient degumming [19,30,31,32,33,34,35,36]. However, the efficacy of this method is compromised by the challenging task of sericin retrieval from the soap solution, rendering the recovery process intricate. The resultant wastewater from the degumming process contains residues of sericin, salts, and soap, necessitating comprehensive purification and prompting the exploration of alternative degumming strategies.

### 4.3. Chemical Extraction Methods

Industrial practices often adopt simplified approaches, such as degumming through the use of various chemical treatments, which circumvent the use of expensive additives like Marseille soaps. Acids (citric, tartaric, succinic, etc.) or bases (sodium carbonate, sodium phosphate, sodium silicate, sodium hydrosulfite, etc.) are used to extract the sericin from silk. These chemicals hydrolyze sericin by breaking the peptide bonds between amino acids, resulting in the release of sericin into an alkaline or acidic solution, where it is highly soluble [37,38,39,40]. The use of acids and bases to extract sericin can significantly degrade the protein [38]. On the contrary, the urea degradation extraction method, often supplemented with 2-mercaptoethanol, demonstrates a reduced degradative impact on sericin. This approach allows for the extraction of approximately 95% of the total sericin content within the fiber without inducing damage. However, despite its efficacy, this method is characterized by its high cost and time-consuming nature [39]. Additionally, sericin obtained through urea-based extraction exhibits high toxicity toward cells [41]. Despite some advancements, challenges persist in recovering high-quality sericin due to the residual chemical impurities inherent in the degumming process [13]. Moreover, the environmental ramifications of these methodologies warrant attention, as the presence of chemical remnants in residual water compromises ecological integrity [13].

### 4.4. Biological Extraction Methods

Alternatively, enzymatic approaches have emerged as a favorable strategy for sericin degumming, primarily due to their inherent efficiency in energy consumption [19,34,42,43]. The utilization of enzymes in degumming is connected to the discovery of cocoonase, a class of proteinases adept at breaking down sericin bindings. Various enzymes such as trypsin, papain, and bacterial enzymes have been prominently employed for the degumming process [44]. Trypsin, a proteolytic enzyme, targets the peptide bonds between the carboxyl group of lysine or arginine and adjacent amino acids, a process facilitated by sericin’s elevated lysine and arginine content. Papain, with its broad specificity toward polypeptides, also serves as an effective agent for cocoon degumming [45]. Furthermore, alcalase, a bacterial enzyme, along with several fungal protease enzymes, have been standardized and proven economically viable, devoid of chemical hazards [44]. The concentration of enzymes and duration of treatment significantly impact the process kinetics. Additionally, the chemical properties of soluble sericin peptides vary depending on the enzyme employed. Although marginally pricier than the aforementioned techniques, this method demands less energy, rendering it more environmentally sustainable [35]. The concurrent utilization of the enzymes savinase and alcalase, along with ultrasound, has also been explored for sericin extraction from silk fibers [28]. This approach enhances the efficacy of the degumming process with prolonged treatment duration. However, the authors have not examined the integrity of the isolated sericin [28]. Notably, an extracellular protease derived from *Bacillus* sp. exhibits remarkable specificity in sericin removal under mildly alkaline pH conditions. While the fibrous structure remains unaltered, sericin undergoes degradation into peptides measuring 10–12 kDa in size, underlining the selective action of protease enzymes in degumming processes [43]. Similarly, a recently isolated thermostable alkaline serine protease from *Bacillus halodurans* displays superior degumming efficacy compared to commercial alcalase protease [46].

### 4.5. Thermal Extraction Methods

Furthermore, thermal extraction primarily involved boiling cocoons in hot water, a method appreciated for its straightforwardness and minimal chemical intervention [8]. The utilization of hot water, typically between 80 °C and 100 °C under atmospheric pressure, has garnered significant attention, while the development of high-pressure techniques, facilitated by lab-scale autoclave machinery, has further expanded the horizons of silk degumming methodologies [13,47,48]. Regardless of the specific technique employed, a common assurance prevails—extraction of sericin by boiling in water under ambient or increased pressure offers the distinct advantage of ensuring the absence of impurities and direct use of recovered sericin without dialysis [13]. While thermal extraction may result in certain levels of sericin degradation, particularly with elevated temperatures or prolonged exposure, sericin maintains its exceptional properties, rendering it the most widely employed approach [49]. Variations in extraction conditions, such as temperature, pressure, and heating duration, play a significant role in determining the molecular weight of extracted sericin from cocoons. In essence, these conditions offer a means to regulate the molecular weight of sericin during extraction [42].

### 4.6. Modern Extraction Methods

Emerging technologies have been devised to extract sericin from fibroin in a more environmentally friendly, efficient, and sustainable manner. These include methods utilizing infrared heat, microwave, steam treatment, carbon dioxide supercritical fluid, and ultrasonication [16,31,35,42,47,48,49,50]. Explorations into those treatments have offered promising avenues for reducing water consumption during extraction, aligning with sustainability objectives. However, it is worth noting that the resulting sericin may exhibit variances in molecular weights contingent upon extraction parameters such as temperature and duration [47]. A recent breakthrough study showcased the efficacy of infrared heating in achieving complete removal of sericin from raw silk, yielding a protein content of superior quality compared to conventional methods [47]. This innovative approach leveraged radiation heating, which directly transferred energy to the material through electromagnetic waves, thereby facilitating sericin detachment and enhancing solubility in water. In this process, water molecules act as abrasives during energy transfer by electromagnetic waves, thereby enhancing the sericin shedding. Further on, findings from one study [16] suggested that microwave degumming offers several advantages, including short extraction time, low energy consumption, and absence of chemical pollution. In comparison to other degumming methods like acid and alkaline-based, boiling, high-temperature, and high-pressure, microwave degumming emerges as the most effective technique together with infrared heating. Since infrared and microwave heating eliminate the need for additional chemicals in the degumming process, their solutions are conducive to achieving high-purity and cost-effective extraction of sericin. Furthermore, spectroscopic analyses revealed that infrared extraction methods resulted in minimal denaturation and degradation of sericin molecules compared to conventional autoclave extraction techniques, underscoring the potential of infrared heating in mitigating protein degradation [47]. Steam treatment effectively removes sericin from silk fibers using pressurized steam without any chemicals, minimizing water pollution and consumption, maintaining desirable physicochemical properties, and being more energy-efficient and economical compared to conventional methods [35]. Moreover, the carbon dioxide supercritical fluid method offers an environmentally stable alternative to conventional extraction methods, effectively cleanly removing sericin protein [50]. Similarly, ultrasonic extraction was proven to efficiently and environmentally remove sericin from fibroin, enhancing degumming rate and fiber quality, particularly at lower temperatures, compared to conventional thermal methods [42].

### 4.7. Sericin Extraction: Impact on Environment, Economy, and Functionality

Each extraction method has distinct advantages and limitations, as summarized in Table 5. Acidic and basic extraction methods, while effective, significantly degrade the sericin structure, resulting in a loss of functionality and requiring further purification to remove chemical residues [38], as discussed in Section 4.3. Additionally, these methods raise environmental concerns due to the toxic waste generated, necessitating careful disposal, thereby increasing costs and impacting sustainability. Urea-based extraction methods are time-consuming and costly, as noted in Section 4.3, and they pose environmental challenges due to the toxicity of urea and the hazardous waste produced [39]. In contrast, thermal extraction using hot distilled water is the most employed method due to its simplicity and lower environmental impact. This approach is highlighted in Section 4.6, where boiling cocoons in water are noted for their minimal chemical intervention and the ability to produce sericin free from impurities [8]. Although it may cause some protein degradation, especially at high temperatures or with prolonged exposure [49], the absence of chemical additives makes this method economically feasible and scalable, leading to its wide acceptance in industrial applications. Recently, more sustainable techniques, such as infrared heating, supercritical CO_2_ extraction, and ultrasound extraction, have been explored [31,42]. These methods reduce reliance on harmful chemicals, enhancing their eco-friendliness. However, as noted in Section 4.5, they require specialized equipment, which increases initial costs and may hinder widespread adoption until their economic viability improves. To explore the functional characteristics of sericin, it is important to examine the conformational changes in its secondary structure that result from various extraction methods. Conventional and alkali-degradation extraction techniques yield sericin with a composition of α-helices, random coils, and turns. In contrast, sericin extracted through autoclaving lacks α-helical structures [51]. Notably, regardless of the extraction method employed, sericin consistently exhibits a negative zeta potential, with urea-extracted sericin displaying the highest negative charge, followed by acid-degraded, heat-degraded, and alkali-degraded sericin [39]. Research by Aramwit et al. [39] indicates that serine is the most abundant amino acid in sericin across all extraction methods, followed by aspartic acid and glycine. Thermal-extracted sericin shows significantly higher levels of methionine, whereas urea-extracted sericin has notably lower tyrosine content. Furthermore, extraction methods influence the presence of secondary metabolites; for instance, thermal extraction results in higher total phenol content, while urea extraction yields lower levels. Acid-degraded sericin is characterized by the highest flavonoid content, in contrast to the lowest levels found in alkali-degraded sericin [51]. The choice of extraction method significantly affects the properties of sericin, thereby influencing its potential applications. The arrangement of sericin’s amino acids, encompassing aggregation, β-sheet formation, and β-turns, can impact cell behavior. Minimizing chemical degradation is essential for enhancing cell growth and attachment, primarily due to the spatial arrangement of methionine and cysteine residues [5]. Additionally, the amino acid composition of sericin plays a crucial role in its biological properties and performance as a biomaterial. For example, sericin with higher concentrations of serine and threonine demonstrates improved antioxidant and photoprotective activities. Moreover, different extraction methods have been shown to variably impact cell viability and collagen production [39].

## 5. Purification

**Figure 7:**
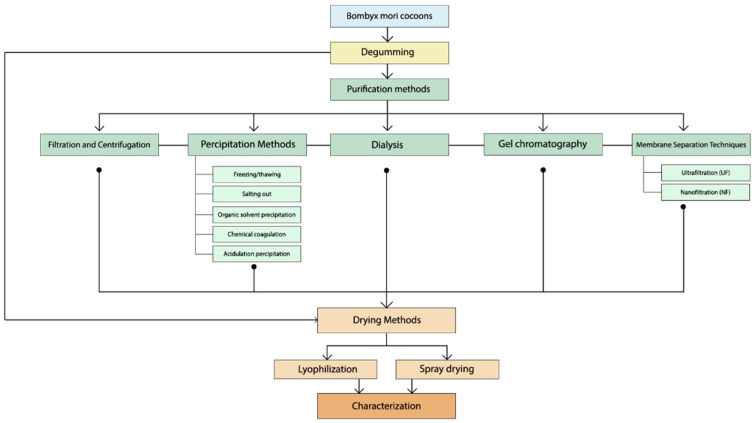
Sericin purification process overview.

After the initial degumming process, further isolation and purification steps are undertaken to obtain purified sericin. Generally, after each degumming process, the extracted sericin solution undergoes paper filtration to be separated from insoluble fibroin, followed by centrifugation to further separate any remaining impurities. Precipitation methods, such as acidulation precipitation, chemical coagulation, organic solvent precipitation, and salting out can also be used, involving the addition of a precipitating agent to induce the sericin protein to separate of solution, or using a freezing/thawing method [52,53,54,55]. Dialysis can be employed if chemical extraction methods are used, particularly those involving sodium carbonate, citric acid, or urea [42,43]. Optionally, gel chromatography can be applied for further purification based on molecular size, followed by membrane filtration to remove smaller impurities [56]. However, precipitation methods pose challenges such as low recovery rates (around 40%) and increased risk of secondary pollution due to the involvement of chemicals [57]. Once isolated, the sericin protein is typically dissolved and the solution needs to be dried to obtain a stable powder or solid form. Drying methods such as lyophilization and spray drying can be used to remove the water from the sericin solution [55,58,59]. Although these techniques are widely utilized depending on the desired level of purification, challenges remain in balancing recovery rates and purity. In response to these limitations, membrane separation techniques, particularly ultrafiltration (UF) and nanofiltration (NF), have emerged as promising alternatives for obtaining higher recovery rates (above 80%) and higher purity of sericin protein [15,33,60]. The steps outlined in Figure 7 enable the effective purification and refinement of sericin, resulting in a high-quality product suitable for various industrial applications. Each stage of the process is thoughtfully designed to maximize yield, enhance purity, and improve efficiency while minimizing the degradation of the sericin protein.

## 6. Biological and Medical Applications

**Figure 8:**
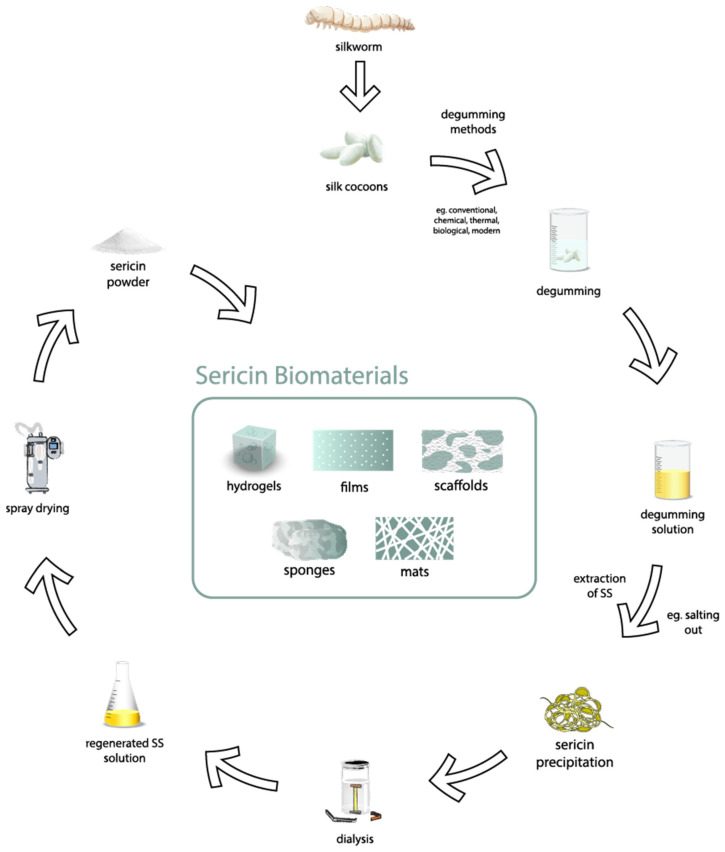
Processing and types of regenerated sericin biomaterials.

As described in Section 3, silk sericin is a versatile biomaterial suitable for various biomedical applications due to its biocompatibility, biodegradability, and multifunctional properties, including anti-inflammatory, antibacterial, antioxidant, anti-tyrosinase, and anti-aging effects. This section will elaborate on these applications in detail. Figure 8 illustrates the comprehensive processing of regenerated sericin biomaterials and highlights the various forms they can take for biomedical applications. Initially, the process involves degumming to extract and dissolve sericin. The degumming solution, containing sericin, is subjected to a salting-out method to precipitate the sericin, which is then dissolved in distilled water to form a sericin solution. This solution undergoes dialysis to yield a regenerated sericin solution, and spray-drying of this regenerated solution produces sericin powder. Currently, silk biomaterials derived from this process are available in various forms, including hydrogels [61,62,63,64,65,66], films [67,68,69,70,71], mats [72], scaffolds [62,73,74,75], sponges [76], and particles [77,78,79,80], showcasing their versatility in biomedical applications.

### 6.1. Drug Delivery Systems

Given their diverse structural features and excellent biocompatibility, silk proteins find extensive applications as delivery systems for a wide range of bioactive molecules, including drugs and various small molecules [81,82]. Sericin, with its amphiphilic nature characterized by both polar side chains and hydrophobic domains, emerges as a versatile vehicle capable of efficiently binding charged molecules [83]. Its prolonged half-life in vivo, attributed to enhanced retention in kidney filtration, coupled with remarkable moisture absorption and desorption abilities, highlights its crucial role in drug delivery [84]. Additionally, sericin’s capacity to expand and contract further enhances its appeal in this context [85]. Whether utilized in its pure state or blended with other polymers, sericin can be customized for a variety of purposes, including the creation of implantable or injectable materials such as matrices, particles, and hydrogels, thereby meeting specific needs and requirements [31]. Several of these recent applications can be found in the accompanying Table 6.

**Table 6:** Recent studies investigating sericin’s role in delivery systems.

Materials	Medical Condition	Cell/Drug Delivered	Refs.
SS/PAC ^a^	Ulcerative Colitis	Proanthocyanidins	[77]
SS@FeS ^b^	Breast Cancer	Nano agent	[78]
			
SS-PLA ^c^	Cancer Therapy	Doxorubicin (DOX)	[86]
SSC-NPs ^d^	Cancer Phototherapy	Chlorin e6 (Ce6)	[87]
MR-SNC ^e^	Breast Cancer	Resveratrol and Melatonin	[88]
Sericin Microparticles- MON	Metastatic Lung Cancer	Doxorubicin (DOX)	
Genipin/sericin hydrogels	Ischemic Stroke	neurotrophic cytokines	
Zein/sericin nanoblends	Antitumor	5-Fluorouracil	[89,90]
SS-NPs ^f^	Cancer Immunotherapy	Doxorubicin (DOX) and Indocyanine green (ICG)	[91]
Cispt-SNC ^g^	Breast Cancer	Cisplatin	[92]
Fucoidan and Sericin	Chronic Inflammatory Diseases	Diclofenac sodium (DS)	[93]

^a^ Silk sericinproanthocyanidins composites, ^b^ Silk sericin–ferric sulfide nanoparticles, ^c^ Silk Sericin–Polylactide, ^d^ Silk sericin-based nanoparticles, ^e^ Melatonin–resveratrol sericin-based nanocarrier, ^f^ Silk sericin-nanoparticles, ^g^ Cisplatin–sericin-based nanocarriers.

Sericin-based structures, primarily hydrogels, are often created through processes like crosslinking, precipitation, chemical modification, or blending with other polymers. These structures have shown promise in drug delivery applications. For instance, hydrogels formed from sericin can serve as matrices for incorporating therapeutic agents, providing sustained release profiles and targeted delivery to specific tissues or cells. Additionally, the versatility of sericin allows for the modification of these structures to enhance their drug-loading capacity, biocompatibility, and stability. Zhang et al. [94] utilized sericin in conjunction with ionically cross-linked alginate to fabricate hydrogels capable of not only hosting myoblast cells but also stimulating their proliferation, migration, and viability. However, monitoring certain aspects of injectable hydrogels in vivo, such as drug release kinetics and gel degradation, presents challenges. To address this, a study by Hardy et al. [81] developed a sericin/dextran injectable hydrogel, which exhibited efficient drug loading and controlled release of both macromolecular (horseradish peroxidase, HRP) and small molecular (antitumor drug doxorubicin, DOX) drugs. Moreover, this hydrogel served as a photoluminescence-trackable drug delivery system, as the sericin’s photoluminescence directly and stably correlated with its degradation. This capability enabled long-term in vivo imaging and real-time monitoring of the remaining drug. Remarkably, a substantial quantity of natural silk sericin was extracted directly from the silk glands of silkworm mutants lacking fibroin in another study conducted by Zhang et al. [94]. This silk sericin (SS) was then crosslinked with H_2_O_2_, resulting in a robust sericin hydrogel characterized by a high elastic modulus. Unexpectedly, SS-H_2_O_2_ (silk sericin crosslinked with H_2_O_2_) exhibited exceptional mechanical resilience and durability, making it a promising candidate for sustained-release drug delivery. Protein-based nanoparticles are highly efficient carriers for the controlled release of pharmaceuticals or cells, as they degrade into non-toxic, absorbable byproducts [95]. The chemical reactivity of sericin enables the production of drug carriers, such as nanoparticles and microparticles, by facilitating the straightforward binding of molecules [31]. In relation to that, one study [79] developed a novel siRNA delivery system for treating laryngeal cancer. By synthesizing albumin–sericin nanoparticles with different ratios of albumin and sericin, researchers addressed the challenge of delivering siRNA effectively. These nanoparticles, modified with poly-L-lysine (PLL) and hyaluronic acid (HA), successfully targeted laryngeal cancer cells and silenced overexpressed genes. The optimized formulation achieved high siRNA entrapment efficiencies and effectively inhibited cell growth and induced apoptosis.

Another study highlighted sericin nanoparticles’ potential as highly efficient carriers for transporting bioactive compounds to specific target cells [80]. Researchers found that silk sericin can be a promising bio-nanocarrier for resveratrol delivery. Resveratrol-loaded sericin nanoparticles (RSV-loaded SP), measuring 200–400 nm in size, with negative charges, were developed. Encapsulation varied with sericin concentration (0.6% and 1.0% *w*/*v*), showing sustained resveratrol release over 72 h. These nanoparticles were non-cytotoxic to the skin, inhibited colorectal adenocarcinoma cell growth, and were internalized by cells. A recent study [77] aimed to expand silk sericin’s role as a drug carrier by developing a simple method for creating sericin-stabilized drug composites. Encapsulating insoluble drugs like proanthocyanidins (PAC) into sericin achieved high drug loading and uniform dispersion in water. The resulting SS/PAC composites showed notable antioxidant effects and biocompatibility, indicating potential for therapeutic use, especially in treating conditions like ulcerative colitis. Another recent study [78] attempted to develop a highly efficient photothermal nanoagent, SS@FeS, for tumor treatment. The nanoagent exhibited excellent water dispersibility, high photothermal ability, and potent anticancer performance by integrating natural sericin protein with ferric sulfide nanoparticles. It efficiently entered cells, accumulating in lysosomes and mitochondria, where it induced enhanced cytotoxicity against 4T1 tumor cells by damaging mitochondria. Additionally, the nano agent demonstrated a fenton reaction, enhancing its photothermal therapy (PTT) efficacy. These findings suggest the potential of SS@FeS as a promising candidate for photothermal agent-based tumor treatment. Moreover, a study [91] in 2023 aimed to use sericin nanoparticles (SDINPs) to induce immunogenic cell death (ICD) in cancer therapy through photothermal therapy (PTT). SDINPs delivered chemotherapeutic drugs and photosensitizers to target cancer cells, showing enhanced therapeutic efficacy and PTT-mediated ICD induction. Due to sericin’s impressive hydrophilicity, hydrophobic entities such as cholesterol [96], polylactide [86], doxorubicin [97], and chlorin e6 [87] can be attached to sericin to form amphiphilic conjugates. These conjugates could spontaneously assemble into micelles, facilitating the delivery of pharmaceutical agents. In addition to its applications in drug and cell delivery, sericin also possesses potential as a contrast agent. It can be linked with specific targeting ligands or imaging probes, facilitating targeted imaging for various therapeutic purposes [5,98]. Sericin’s pH sensitivity stems from its highly polar side groups, including hydroxyl, carboxyl, and amino functionalities. Researchers have extensively explored its potential in developing smart delivery systems, particularly pH-responsive ones. These systems offer notable advantages, allowing for precise control over therapeutic compound release in response to external acidic or alkaline conditions. This specificity enhancement not only improves efficacy but also minimizes potential side effects [90,91]. The hydrazone bond, renowned for its pH-sensitive nature, has frequently been employed in fabricating drug delivery systems based on sericin that responds to acidic conditions [86,88,92,97]. A 2023 study developed a pH-responsive sericin-based nanocarrier (MR-SNC) for co-delivering resveratrol and melatonin to MCF-7 breast cancer cells [88]. Using flash-nanoprecipitation, MR-SNC was designed with various sericin concentrations to exhibit pH-dependent behavior. In vitro studies confirmed significant pH-dependent drug release, cellular uptake, and cytotoxicity, demonstrating its potential for controlled release in acidic environments. Therefore, the study highlighted MR-SNC’s pH-dependent charge reversal property, enhancing drug delivery specificity and efficacy in acidic tumor microenvironments. In addition to the seminal works on this subject [85,97,99,100], there have been subsequent developments inspired by prior research endeavors. The target of another novel investigation [86] was to develop pH-responsive drug delivery materials using sericin as a building block. Amphiphilic substances were synthesized by conjugating hydrophobic polylactide (PLA) with hydrophilic sericin using a bis-aryl hydrazone linker. The pH-dependent drug release from SS-PLA nanoparticles was investigated using doxorubicin (DOX) as a model drug. Results showed that the release rate of DOX was slower at physiological pH (7.4) compared to pH 5.0, indicating pH dependency. Therefore, this investigation emphasized the promising role of sericin-based amphiphilic materials as effective drug carriers for cancer therapy, particularly highlighting their pH-responsive characteristics. A study in 2024 developed pH-sensitive sericin-based nanocarriers (SNCs) for controlled cisplatin delivery in breast cancer therapy [92]. These SNCs, produced via nanoprecipitation, encapsulated cisplatin and displayed a crucial charge-reversal property for effective drug release. Optimizing sericin concentration, they achieved suitable size and high drug encapsulation for efficient cellular uptake. Physicochemical analyses confirmed SNCs and Cispt-SNCs’ suitability for effective drug delivery. Interaction between Cispt-SNCs and cells induces apoptosis, highlighting their potential for targeted therapy.

### 6.2. Tissue Engineering

Given its established efficacy in the cosmetic sector, as outlined in Section 7, silk sericin was first used in skin tissue engineering within biomedical applications. Furthermore, owing to its recognized mitogenic and osteogenic properties, sericin-based biomaterials have broadened their scope to encompass tissue engineering applications in bone, cartilage, cardiac, neural, and muscle tissues. As sericin’s diverse potential emerges, it is progressively used in various areas including inflammatory disorders, bio-adhesion, hemostasis, and beyond. Sericin, when cross-linked with various polymers to form gels, offers versatile applications, particularly in wound healing. Its biocompatibility ensures minimal immunological reactions, distinguishing it from other biomaterials. Among contemporary wound dressings, sericin-based films [67,68,69,70,71], hydrogels [61,62,63,64], and scaffolds [62,73,74,75] are favored choices due to their ability to serve as physical barriers and absorb exudates. The recently reported works on sericin-based wound healing systems are highlighted in Table 7. In the context of films, which serve as highly flexible and elastic structures facilitating gas exchange, water vapor transmission, and bacteria isolation [101], sericin films crosslinked with glutaraldehyde and composite films of sericin/collagen were fabricated. These films aim to facilitate wound healing by promoting the attachment and growth of diverse cell types [69,71]. Also, to impart antibacterial properties to the dressing materials, nanoparticles with antibacterial properties were incorporated into sericin-based films [102,103,104,105]. Embarking on the elucidation of diverse sericin-based hydrogels for wound healing applications, it is essential to note that sericin hydrogel was utilized as an in situ wound healing system demonstrating the potential to regenerate the skin both in vitro and in vivo. The hydrogel exhibited non-toxic properties toward L929 fibroblast cells and facilitated cell adhesion, colonization, and proliferation. In diabetic mice, wounds treated with hydrogel for 21 days displayed decreased granulation tissue and inflammatory cells, as well as a reduction in wound size compared to those treated with a conventional clinic-used dressing. Another study [64] developed hydrogels combining sericin, chitosan, and glycosaminoglycans, supplemented with growth factors to enhance cellular functions and facilitate skin tissue repair. In vivo studies demonstrated the hydrogels’ biocompatibility and effectiveness in promoting skin tissue repair and angiogenesis, with minimal immune response. These hydrogels mimic natural skin tissue properties and provide a conducive environment for skin regeneration. Incorporating antibiotics and other antimicrobial agents into the hydrogels amplifies their antibacterial and antifungal capabilities, addressing the common issue of wound infection during the healing process. Hence, antimicrobial components such as nano-silver particles [65,106], tetracycline [107], and zinc oxide nanoparticles [108] are commonly incorporated into wound dressings based on silk sericin. For instance, in a 2023 study [106], silver ion (Ag+)-modified chitosan (CS) nanoparticles were used to deliver lupeol (L), resulting in the formation of CS-Ag-L-NPs. These nanoparticles were enclosed within a thermosensitive self-assembling sericin hydrogel, offering several advantageous outcomes. These included restraining bacterial proliferation on wound surfaces, accelerating re-epithelialization to enhance wound closure, mitigating inflammation, and fostering the deposition of collagen fibers. Utilizing a rat model with infected wounds, it was observed that sericin hydrogels incorporating CS-Ag-L-NPs continuously released lupeol in vivo, showcasing antimicrobial properties and facilitating wound recovery. Moreover, the development of an oriented SS microneedle through a template method illustrates how rationally designed material formulations can aid in expediting wound healing. In this approach [108], the central needles are relatively shorter in length compared to the edge needles, and this unique structural design facilitates wound closure by contracting the wound edges, thereby physically promoting the healing process. Biomaterial scaffolds are sought to create an instructive environment conducive to cell recruitment and proliferation, thereby expediting healing cascades [109]. Sericin is used in scaffold fabrication through techniques such as foam processing, solvent casting, or freeze-drying techniques. Additionally, a 3D-printed hydrogel scaffold was developed [62] consisting of sericin and methacrylic anhydride-modified gelatin (GelMA) through 3D printing. Their findings showed its ability for skin wound regeneration, with emphasis on the transparency of the hydrogel, facilitating wound visualization. Years later, another research [75] explored a natural dual protein-based nanofibrous scaffold derived from *B. mori* silkworm cocoons, containing silk fibroin and sericin, for wound healing. The scaffold, featuring three layers—a silk fibroin–PVA blend, a sericin layer containing silver(I) sulfadiazine, and a silk fibroin–PCL blend—exhibited notable properties including excellent wettability, controlled drug release, antibacterial, and antioxidant effects. In vivo experiments on male Balb/c mice demonstrated complete wound healing and new tissue formation, indicating its potential as a promising wound dressing material with antibacterial and antioxidant properties.

**Table 7:** Recent studies investigating sericin’s role in wound healing processes.

Materials	Form	Refs.
Fibroin, Sericin, Silver Nanoparticles and Gentamicin	Films	[110]
Silkworms, Sericin	Scaffold	[111]
Polyvinyl alcohol, Sericin, Azithromycin, Genipin	Hydrogel	[66]
Sericin, Chitosan, Silver Nanoparticles	Films	[112]
Sericin, Human Placenta-derived Extracellular Matrix	Scaffold	[113]
Gellan gum, Sericin, Halloysite nanotubes encapsulated with Polydopamine	Hydrogel	[114]
Carboxymethyl Chitosan, Sericin–Silver nanoparticles, Halloysite	Sponge	[115]
Sericin, Polyvinyl alcohol, *Moringa oleifera* leaves extract	Hydrogel	[116]
Sericin, *Jasminum grandiflorum* L. leaves extract	Cream	[103]
Sericin, heparin, basic fibroblast growth factor (bFGF)	Hydrogel	[117]

Sericin presents inherent osteogenic stimulation potential, rendering sericin-derived biomaterials advantageous for bone repair applications. This was confirmed in a study conducted in 2022 [102], where sericin extract enhanced osteoblast cell proliferation and showed antibacterial activity against *Staphylococcus aureus*, reducing biofilm formation by up to 95%. Sericin with normal saline exhibited higher stability and smaller particle size. To regenerate bone tissue effectively, blending sericin with hydroxyapatite or other calcium phosphate-based materials is common practice, as it mimics both the organic and inorganic components of bone matrices. Calcium phosphate (CaP) is a substance widely utilized for bone grafting and offers chemical stability, biocompatibility, low density, and crystallinity [118]. Various studies have explored composite materials combining calcium phosphate with proteins, synthetic polymers, or natural polymers to emulate bone tissue quality. While synthetic polymers may lead to local reactions due to monomer release during polymer degradation, natural polymers can yield diverse products due to differences in raw material characteristics [119]. In this regard, sericin/CaP emerges as a crucial component, offering nontoxicity, mechanical stability, and a structured self-assembly capability, as sericin facilitates osteoblast migration, attachment, and proliferation, supporting bone formation. This approach addresses sericin’s inherent mechanical limitations, ensuring scaffolds with suitable mechanical properties for bone regeneration [120,121]. The sericin/CaP composite exhibits potential for antitumor therapy due to its efficient loading and release abilities [122]. These composites possess a notable surface area-to-volume ratio, facilitating biomolecule loading and cellular membrane permeability [123]. Recently, a study [124] aimed to enhance periodontal bone regeneration by developing a guided tissue regeneration (GTR) membrane incorporating sericin–hydroxyapatite (Ser-HAP) composite nanomaterials, which demonstrated potential for periodontal regeneration therapy by promoting osteogenic differentiation of human periodontal membrane stem cells (hPDLSCs). Besides hydroxyapatite, sericin can be combined with alternative biomaterials to enhance its therapeutic efficacy, including growth factors [125] and functionalized agents such as graphene oxide [104]. In relation to that, one of the studies [126] introduced a photo-crosslinked sericin methacryloyl (SerMA)/graphene oxide (GO) hydrogel (SMH/GO) for bone repair. The incorporation of graphene oxide substantially enhanced the compressive strength of a sericin hydrogel formed through photo-polymerization, owing to its osteoinductive and mechanical characteristics. A year later, an injectable hydrogel for bone regeneration, comprising alginate, sericin, and graphene oxide, was developed [104]. Synergistically, graphene oxide enhanced cell spreading and stimulated the osteogenic differentiation of bone marrow-derived mesenchymal stem cells (BMSCs) by increasing the expression of genes associated with osteogenesis cells. Both the sericin hydrogel and sericin–alginate composite hydrogel significantly enhanced bone regeneration in rat calvarial and femur defect models, respectively. The sericin hydrogel facilitated BMSC migration and osteogenic differentiation by activating mitogen-activated protein kinases, tumor necrosis factor (TNF), and chemokine signaling pathways for bone regeneration [126]. On the other hand, the sericin–alginate composite hydrogel indirectly influenced osteogenic differentiation by promoting M2 macrophage polarization. This effect was facilitated by sericin, which activated signaling pathways leading to M2 macrophage polarization and subsequent osteogenic differentiation [104]. Additionally, in vivo experiments have shown that sericin can enhance alkaline phosphatase activity, an enzyme crucial for bone tissue mineralization [105]. Functionalized with collagen–fibrin scaffolds, silk sericin accelerated mineralization, enhancing osteoblastic differentiation and matrix mineralization, demonstrated by alkaline phosphatase activity measurements [127]. These hybrid gel scaffolds, created through automated gel aspiration ejections, can be customized for specific properties, serving as a model to study cell-matrix interactions for bioengineering purposes. In addition to applications in repairing and regenerating tissues, sericin-based biomaterials have been utilized to enhance hemostasis [128] and tissue adhesion [129,130]. Furthermore, they demonstrate effectiveness in treating oxidative stress and inflammatory diseases [129,130,131,132]. For nerve tissue engineering, sericin-based hydrogels and scaffolds have shown promise in repairing peripheral nerve injuries [133,134,135,136,137] and treating ischemic stroke [90,131,132]. A nerve guidance conduit (NGC) is typically designed as a hollow tubular structure made from natural or synthetic materials, providing an alternative to grafted nerve tissue for the repair of peripheral nerve injuries [133]. Owing to its excellent biocompatibility and biodegradability, sericin has been utilized for peripheral nerve repair in combination with silicone [134]. This sericin/silicone conduit effectively facilitated the restoration of nerve structure and function while repairing a 5 mm gap defect in a sciatic nerve transection model. To enhance repair efficiency, a sericin nerve conduit loaded with clobetasol, a glucocorticoid receptor agonist, was used to treat a 10 mm nerve defect. The clobetasol-loaded conduit effectively repaired the defect in rats by promoting neurotrophic factor secretion and upregulating myelin-related genes in Schwann cells [135]. Moreover, it was revealed that the degraded components of a genipin-crosslinked sericin hydrogel could facilitate axon extension and branching, as well as protect neurons from hypoxia-induced cellular death [131]. Combining sericin with nerve-stimulating molecules like nerve growth factor [136] and carbon nanotubes [132] has resulted in synergistic effects. Furthermore, sericin-based biomaterials have proven effective in repairing injuries to skeletal muscle [137], addressing ischemic myocardial infarction [138], and facilitating recovery from uterine damage [139,140]. These diverse applications underscore the potential of sericin as both a therapeutic agent and a biomaterial.

### 6.3. Other Applications

The utilization of sericin as a supplement in culture media was initially reported in 2002 [141]. It has been demonstrated that sericin emerges as a promising substitute for bovine serum albumin (BSA), traditionally used to enhance serum-free cell culture media. Sericin boosted cell proliferation across different mammalian cell lines, including hybridoma 2E3-O, human hepatoblastoma (HepG2), human epithelial (HeLa), and human embryonal kidney (293) cells, showing similar effectiveness to BSA and even enhanced proliferation when combined with it [141]. Additionally, it showed the ability to enhance the proliferation of human skin fibroblast cells, including Balb3T3 cells and RC4 corneal cells [142]. Unlike BSA, sericin retained its activity after autoclaving, suggesting it could be a safer alternative for stimulating cell growth [141]. Moreover, insect-derived sericin offers potential advantages over animal-derived supplements like BSA due to its safety profile [141]. Therefore, sericin presents distinct advantages—it is animal-free, sustainably sourced, and biocompatible. Two recent studies investigated the effect of sericin supplementation on bovine oocyte nuclear maturation [143,144], with one study [143] also assessing DNA fragmentation. Both studies concluded that supplementation of 0.1% sericin enhanced nuclear maturation, and one study [143] further noted improvements in oocyte nuclear status while maintaining DNA integrity. In contrast to previous studies on bovine oocytes, research by Tial et al. [145] aimed to boost the developmental potential of prepubertal lamb oocytes for juvenile in vitro embryo transfer (JIVET). By supplementing the in vitro maturation (IVM) medium with antioxidants and cytokines, including 0.5% sericin, significant improvements in blastocyst rates were observed. Furthermore, the addition of fibroblast growth factor 2 (FGF2)-leukemia inhibitory factor (LIF)-insulin-like growth factor1 (IGF1) (FLI) significantly enhanced blastocyst development. Notably, the developmental competence achieved with sericin and FLI rivaled that of adult follicular fluid, suggesting promising avenues for improving JIVET outcomes. Another study in 2022 [141] assessed whether sericin could enhance flavivirus amplification in cell cultures, particularly Zika virus (ZIKV). The results showed that adding sericin at 80 µg/mL significantly boosted ZIKV production in both insect (C6/36) and mammalian (Vero) cell lines, increasing infectious particle concentrations by 1 log. Sericin also demonstrated cryoprotective properties for C6/36 cells. Turning attention to human spermatozoa, the influence of sericin supplementation on human sperm cryopreservation was investigated [146]. Results demonstrated that adding sericin to both freezing and thawing media significantly improved sperm viability, and motility, and reduced DNA fragmentation, suggesting its potential as a cryoprotective supplement for human sperm cryopreservation. Several recent studies explored the effects of sericin supplementation on different biological systems, promoting viability, maturation, and pigmentation of human fetal and adult retinal pigment epithelial cells [147], enhancing in vitro maturation rates of mouse embryos [148], and improving post-thawed quality of boar semen [149]. Metabolic dysfunctions such as diabetes and hypercholesterolemia are associated with chronic inflammation, necessitating effective treatments with minimal side effects [150]. There is an increasing demand for safer antidiabetic agents due to the limitations of current therapies [151]. Emerging evidence underscores the hypoglycemic [152,153,154] and hypocholesterolemic properties [155,156,157,158] of sericin. Regarding anti-diabetes, one of the studies [154] evaluated the hypoglycemic effects of orally administered sericin protein in type 2 diabetic mice. The results showed reductions in blood glucose levels, improved insulin sensitivity, and enhanced antioxidative activity, indicating sericin’s potential in managing diabetes and inflammation. Two years later, another study [152] investigated the impact of degraded sericin on liver injury in type 2 diabetic rats. Dietary supplementation with sericin improved liver health and reduced inflammation, suggesting its potential as a functional food for managing blood sugar levels. Additionally, sericin can potentially activate the insulin–phosphoinositide 3-kinase/protein kinase B (insulin-PI3K/AKT) signaling pathway, promoting glycogen synthesis, accelerated glycolysis, and inhibited gluconeogenesis [152,159]. As oxidative stress is implicated in hypercholesterolemia, sericin, known for its antioxidative properties, has been shown to reduce serum cholesterol levels in various rat models, mitigating lipid peroxidation and decreasing the absorption of dietary cholesterol in the intestines [155]. Mitochondria play a significant role in ROS production, and the investigation into the effects of sericin administration on mitochondrial architecture integrity showed a decrease in ROS production in hepatic mitochondria [160]. Additionally, sericin was found to upregulate the expression of two antioxidative proteins in hepatic mitochondria through proteomic analysis [157]. A recent study [157] using hypercholesterolaemic rats examined the effects of sericin treatment on cardiac mitochondrial structure and protein expression. The results showed that sericin treatment led to improvements in mitochondrial structure and metabolism and alterations in the expression of key mitochondrial proteins associated with energy production and apoptosis regulation. Therefore, sericin is suggested as a potential treatment for cardiac mitochondrial abnormalities under hypercholesterolaemic conditions.

### 6.4. Challenges and Limitations

Despite its numerous benefits and potential applications, silk sericin faces several limitations that restrict its use in biomedical fields. A key factor is the choice of extraction method, which plays a crucial role in maintaining a consistent physicochemical profile and optimizing biological performance. This method must also be sustainable and scalable for industrial applications, all while preserving the economic viability of the silk industry. Furthermore, the extraction process can significantly influence sericin’s bioactivity [161]. The complexity of sericin, derived from multiple ser genes, further complicates our understanding of its biological activity mechanisms [162]. The limited bioactivity of silk sericin hinders its ability to promote key functions like cellular adhesion and proliferation, which are essential for tissue engineering. To overcome this, sericin often requires the incorporation of bioactive molecules or other polymers to enhance its biological functionality. Addressing these challenges is critical to expanding sericin’s applications in drug delivery, wound healing, and tissue scaffolding. While the current research is promising, several key areas remain unexplored. For instance, more studies are needed to clarify how sericin supports bone cell proliferation and healing, evaluate its long-term biocompatibility in humans, assess the risks of allergic reactions or immune responses, and optimize its integration into graft materials. In addition, nano-formulations of sericin have garnered increasing attention for tissue engineering, drug delivery, and pharmaceutical applications. However, more in vivo research and clinical trials are still required to fully explore the potential of sericin-based nanocomposites in biomedicine. The underlying molecular mechanisms of sericin’s effects also remain unclear, requiring further investigation into cellular interactions to enhance its therapeutic properties through integration with other bioactive agents. This can be achieved through a combination of in vitro and in vivo studies, molecular docking analyses, pharmacokinetics, and proteomic investigations. Moreover, sericin’s high solubility in water and its animal-derived origin can limit its application in specific biomedical areas, especially those prioritizing synthetic or plant-based materials due to concerns about biocompatibility and ethical sourcing [163]. Lastly, sericin’s poor rheological and mechanical properties, including low mechanical strength and elasticity, prevent it from functioning independently as drug delivery systems or scaffolds for tissue engineering. These limitations make it unsuitable for applications that require structural support, such as bone or cartilage regeneration. As a result, sericin-based scaffolds typically need to be combined with other materials to enhance their mechanical properties and overall performance [164]. 

## 7. Industrial and Commercial Applications

### 7.1. Textile Industry Impact

Sericin, an incidental product of silk production, emerges during the degumming process when water-containing sericin is released following the extraction of fibroin fibers. Unfortunately, this discharge poses environmental concerns due to the substantial oxygen demand during sericin’s bacterial degradation [165]. Consequently, optimizing sericin utilization offers a twofold advantage: addressing textile industry waste while capitalizing on its inherent biocompatibility, antibacterial, antimicrobial, and wound-healing properties for textile applications [165]. When applied to fiber surfaces, sericin exhibits numerous advantages. It has been observed to enhance electrical resistance, water retention, water absorption, antibacterial capabilities, and reduce skin irritation. In conjunction with polyester, sericin proves effective in diminishing hydrophobicity, enhancing UV protection, and improving radical scavenging behavior [166]. Belhaj Khalifa et al. studied the effects of applying sericin to wool and cotton fabrics. The results indicated that sericin does not have an affinity toward cotton but does toward wool. Indeed, sericin improved the feel of wool fabrics, along with their absorption capacity. They concluded that sericin could serve as an alternative finishing agent to the toxic ones currently used in the industry. However, the challenge they faced was the short exhaustion rate, and they suggested grafting and crosslinking as alternatives for this treatment [167]. Bhandari et al. optimized the sericin treatment conditions for the dyeability of cotton and concluded that treating cotton with 0.5% sericin, 4% citric acid, and 1% sodium hypophosphite, followed by drying at 70 °C for 4 min and curing at 160 °C for 2 min, was the optimal approach for enhancing dye absorption and color strength. Additionally, they proposed using sericin as an alternative to metallic mordants in dyeing cotton fabrics to reduce water pollution and impart functional properties like antimicrobial and ultraviolet protection properties [168]. A novel eco-friendly reactive dyeing method, employing sericin pre-treatment of cotton fabric, demonstrated a significant reduction in sodium chloride (NaCl) consumption. This approach, validated by Fourier Transform Infrared Spectroscopy (FT-IR) analysis confirming sericin application without crosslinking, yielded comparable color yields and superior washing and rubbing fastnesses, while enhancing crease recovery angles, suggesting sericin’s potential as a pre-dyeing agent to mitigate environmental impact in textile coloring processes [169]. Another study demonstrated that sericin-coated fabric exhibited improved wicking and moisture regain properties, rendering it suitable for direct contact with the dermal layer, particularly for patients with skin diseases. The treated fabric showed enhancements in antistatic, ROS scavenging, and UV absorption attributes, suggesting potential applications in skin moisturizing, cell healing, and anti-aging [170]. In a study investigating the application of Sericin/β-cyclodextrin in skincare textiles, treated cotton samples exhibited excellent scavenging activity and enhanced stiffness, indicating potential applications in cosmetotextiles for sustained skincare benefits post-washes [171]. Sericin plays a crucial role in enhancing the flame retardancy of silk fibers, particularly when combined with metal ions. Research shows that silk fabrics treated with metal ions produce less smoke, have a higher limiting oxygen index (LOI), and form more char, indicating improved fire resistance and wash durability. Sericin not only promotes char formation but also works effectively with metal ions, underscoring its importance in boosting the flame retardancy of silk fabrics, making them more suitable for applications requiring fire safety and long-lasting performance [172].

### 7.2. Food Packaging and Nutraceuticals

The food industry has increasingly turned its attention to packaging solutions, seeking alternatives that address concerns surrounding current packaging methods [173]. A pivotal focus of research lies in identifying a novel coating that is not only cost-effective but also edible, effectively shielding food products from the deleterious effects of oxygen, carbon dioxide, and moisture ingress [174]. Such a coating would serve as a formidable barrier against oxidation, moisture loss, and respiration, thereby extending the shelf life of packaged goods [174]. Sericin, distinguished by its exceptional biocompatibility and biodegradability, presents a promising candidate for functionalizing food packaging materials [175]. Despite its advantageous properties, sericin is abundantly discarded as a by-product within the silk industry [166]. Therefore, leveraging sericin within the realm of food packaging offers a twofold advantage, mitigating waste while concurrently enhancing packaging performance and sustainability within the food sector [166]. Indeed, the global population is increasing constantly and is expected to reach 9.7 billion people by 2050 [176]. This growth imposes greater demands on food production, necessitating increased water, land, and energy resources [177]. Meanwhile, nearly 30% of food is wasted, highlighting the urgency to extend the shelf-life of food products as a viable solution to mitigate such waste [178]. The current coatings, primarily composed of synthetic polymers, significantly contribute to environmental pollution due to their non-biodegradable nature [173]. Natural biopolymers emerge as ideal alternatives [179], with protein-based materials offering biodegradability, renewability, and non-toxicity [180]. Among these alternatives, sericin demonstrates considerable potential for enhancing food quality and extending shelf life [181]. However, utilizing sericin for food packaging encounters challenges such as weak mechanical properties and hydrophilicity [182], which can be addressed by combining sericin with other materials to improve its properties [183]. Several studies have highlighted the promising role of sericin-based edible coatings in extending the storage life of fruits and vegetables. More importantly, the FDA has approved sericin and its derivative as a safe material not causing allergies when taken orally and as having no cytotoxicity effects as an ingredient in cosmetics [184]. Since sericin on its own cannot serve as an effective film for food packaging due to the associated limitations [182], several studies have been performed, highlighting the importance of adding other materials to sericin to ameliorate its properties and make it a suitable candidate for food packaging, as shown in Table 8. To tackle the challenges associated with utilizing sericin as a component of an edible film for food packaging, several studies have been conducted. One primary issue is the weak mechanical properties of sericin, which promote self-aggregation, thus limiting its effectiveness as a packaging material. To address this, nanocellulose, such as bamboo-derived cellulose nanofibrils, can be incorporated to strengthen sericin films [183]. Additionally, combining sericin with other biopolymers can minimize film permeability, enhancing flexibility while reducing the need for plasticizers [8]. Furthermore, chemical crosslinking reactions between sericin and glucose offer a solution to overcome limitations in water resistance and mechanical properties [185]. Secondly, sericin’s hydrophilicity renders it delicate in water environments, affecting its performance. However, incorporating sericin hydrolysate into films can increase water vapor permeability, providing a potential solution [186]. Moreover, sericin films combined with glucomannan and glycerol exhibit improved solubility and flexibility without compromising vapor permeability [8]. Moreover, in terms of preservation, sericin-based edible coatings, containing ingredients like chitosan, aloe vera, and glycerol, show promise in extending the storage life of perishable foods like tomatoes [181]. Additionally, applying glucose to create a sericin coating helps control food oxidation, thereby extending shelf life [185]. These innovative solutions demonstrate the potential of sericin-based materials in addressing key challenges in food packaging, paving the way for sustainable and effective packaging solutions in the industry. Dietary sericin is a sericin protein used as a food ingredient, which has shown exciting potential for improving food products, especially when combined with whey protein. Research indicates that a mere 0.1% addition of sericin to whey protein significantly enhances its mechanical strength, attributed to the formation of hydrogen bonds between the two components [182]. Furthermore, dietary sericin has been effective in reducing serum cholesterol and triglyceride levels, particularly by lowering very low-density lipoprotein (VLDL) levels while maintaining high-density lipoprotein (HDL) levels, potentially reducing the risk of atherosclerosis [187]. Moreover, a patent (Patent No. CN103918853A) describes the application of sericin peptides as an ingredient in the formulation of low-sugar probiotic dextrose candy, specifically designed for individuals with diabetes. Additionally, Mei et al. developed edible bio-nanocomposite films utilizing sericin-derived carbon dots (CDs) and chitosan, providing a multifunctional solution for food preservation. These films exhibit various properties, including anti-counterfeiting, antibacterial, antioxidant, and UV shielding capabilities, highlighting their potential for environmentally friendly food packaging applications [188].

**Table 8:** Biomaterials used with sericin, the respective experimental methods for the preparation, and the resulting added benefits on the properties of sericin.

Biomaterial	Experimental Method	Added Benefit	References
Glucose	Crosslinking	Overcome limitations in water resistance. Improve mechanical properties.	[185]
Glucomannan	Casting	Improve solubility and flexibility.	[189]
Glucomannan and glycerol	Casting	Improve solubility and flexibility. Increase water vapor permeability.	[189]
Chitosan and aloe vera	Casting	Edible food packaging films.	[181]
Bacterial cellulose	Solution impregnation	Improve water intake.	[190]
Nano-cellulose	Casting	Improve the mechanical properties.	[183]
Glycerol	Casting	Enhancement of elongation properties and increase in moisture content.	[191]
ZnONPs and AgNPs on sericin-agarose films ^a^	Casting	Improve water absorption. Enhance mechanical properties.	[192]

^a^ ZnONPs: zinc oxide nanoparticules. AgNPs: silver nanoparticules.

### 7.3. Cosmetics and Skincare Products

Silk-based cosmetics trace their origins back to the 1960s with the production of silk cream, initially incorporating fibroin, but recent focus has shifted toward leveraging the beneficial properties of sericin. Sheng et al. reported that sericin shares properties akin to collagen, a common ingredient in skincare products, known for its resemblance to animal glue, as collagen serves as a primary raw material in skincare cosmetics [193]. Sericin demonstrates exceptional efficacy in moisturizing and whitening [193]. According to the results of their study, sericin exhibited high moisturizing effectiveness because its amino-acid composition is closely similar to that of the natural moisturizing factor (NMF), which is contained in the stratum corneum that retains water and makes the skin plump and elastic. Also, they reported that many polar groups of sericin’s polypeptide chains were on the surface, containing almost the same amount of serine in its chain as the NMF, 29.34% and 30%, respectively [193]. A comparative analysis of moisture absorption showed that a solution containing just 3% sericin achieved the same level of hygroscopicity as a solution with 60% glycerol. This means that sericin can retain moisture effectively at much lower concentrations compared to glycerol. Unlike glycerol, whose excessive use can clog pores and cause irritation due to its molecular weight, sericin’s lower molecular weight allows for easier skin absorption, aiding in reducing localized fine wrinkles [193]. Furthermore, significant absorption of sericin by hair was observed, enhancing hair elasticity and strength [193]. Additionally, a 1% sericin concentration reduced tyrosine activity by over half, thereby inhibiting melanin production and promoting skin whitening [193]. Singh et al. synthesized sericin/β-Cyclodextrin material for skin care finishing on cotton fabric, highlighting promising attributes including antioxidant properties, UV resistance, and effective moisture transmission [194]. Skin-lightening cosmetic products rely on the tyrosinase inhibitors present, which limit the role of tyrosine in producing melanin, and sericin has proven to have strong tyrosinase inhibition [195]. Kim et al. studied sericin as a dietary component and concluded that adding 1% of sericin to the diet for 10 weeks improved epidermal hydration [196]. A patent (Patent No. EP1632214A1) that outlines nail cosmetics with sericin showed that a concentration ranging from 0.02% to 20% has been shown to prevent chapping and brittleness while enhancing the natural glossiness of nails. According to the forecast market value (2032F), the global sericin market is provisioned to reach USD 537.6 million by the end of 2032 [197]. Silk sericin has been used in the development of several cosmetic products for its wide range of benefits for the skin and the hair. Several brands have developed cosmetic products containing sericin, taking advantage of its natural moisturizing effect. Table 9 states six examples of distinct brands utilizing sericin to manufacture products for various applications.

**Table 9:** Sericin products from different brands.

Company	Product Type Example	References
Cicago (Englewood Cliffs, NJ, USA)	Facial moisturizer	EWG [198]
Drunk Elephant (Houston, TX, USA)	Moisturizing shampoo	EWG [198]
Imersa (Denver, CO, USA)	Moisturizing cream	Imersa [199]
Benefit (San Francisco, CA, USA)	Mascara	EWG [198]
Fondonatura (San Donato di Lecce, Italy)	Hair smotherer	Fondonatura [200]
J. And. C. (Como, Italy)	Facial cream	J&C [201]

## 8. Discussions and Future Perspectives

In a world where anthropogenic mass now exceeds the weight of all global living biomass, it is imperative to move away from the traditional linear economy model, which often leads to resource dispersion [202]. This is especially relevant in industries where by-products, such as sericin, retain specific intrinsic properties. Promoting sericin in research is vital due to its potential to address key challenges in biomedicine, materials science, and sustainability. Sericin offers significant advantages over other biomaterials, including excellent biocompatibility, biodegradability, and a wide range of multifunctional properties, such as antioxidant, anti-inflammatory, and antimicrobial effects. These attributes make it particularly valuable for various applications. Moreover, as a versatile globular protein, sericin can be easily functionalized by chemical modifications or crosslinking with other natural or synthetic polymers, enhancing its adaptability across different fields. This functionalization capability distinguishes sericin from many other natural biomaterials, making it both unique and highly versatile. Despite its numerous advantages, sericin has certain limitations compared to other biomaterials. One significant challenge is its economic viability; the extraction and purification processes can be complex and costly. Additionally, sericin exhibits relatively low mechanical strength, particularly when compared to materials like silk fibroin or synthetic polymers. This limitation restricts its use in load-bearing applications. These factors need to be carefully considered when developing sericin-based products, especially in fields that demand high structural integrity or cost-efficiency. Nevertheless, sericin’s unique combination of properties and functional versatility positions it as a highly promising biomaterial for a wide range of applications.

**Figure 9:**
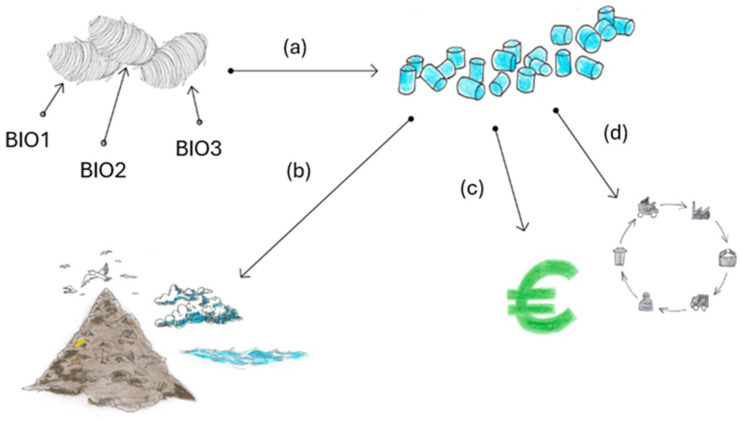
Evaluation of sericin-infused bioplastic pellets for diverse applications; (**a**) Pellets production from silk sericin with other biomaterials, namely BIO1, BIO2, and BIO3; (**b**) Environmental, (**c**) economic, and (**d**) life cycle, assessments of the use of sericin-containing pellets.

The petroleum-based plastic market presents an opportunity for the incorporation of sericin and other bio-based materials, which can be modified to create new blends that have the potential to replace synthetic plastics in the future. Currently, the mass production of sericin, at 0.05 million tons annually [203], is minimal compared to traditional plastics, as sericin is primarily used as an additive in various materials. As shown in Figure 8, sericin can be combined with different biomaterials to produce pellets with enhanced properties, potentially paving the way for its use in diverse fields to replace petroleum-based plastics. Figure 9 displays a representative scheme outlining future perspectives for employing sericin, emphasizing the need for extensive research on its environmental, economic, and life cycle impacts. This research is crucial for understanding the broader effects of utilizing sericin across different applications. Given that approximately 400 million tons of plastics are produced annually [204], capturing even 10% of that market for sericin can bring substantial environmental and economic benefits. As the global population continues to grow, the strain on resources will intensify, highlighting the importance of shifting from a traditional linear economy to a sustainable circular economy. A circular economy prioritizes reusing by-products instead of disposing of them, helping to mitigate environmental consequences while optimizing natural resource consumption. Ultimately, further research on sericin, along with the exploration of its wide-ranging applications, will be crucial for advancing sustainable practices across various industries, addressing resource depletion, and fostering innovative solutions for the future.

## Figures and Tables

**Table 1:** Types and numbers of published studies on sericin in online libraries.

Libraries	PubMed		Science Direct		SpringerLink
Articles	1057	Research articles	2759	Articles	1647
Full Texts	1001	Book chapters	785	Research articles	1279
Associated Data	288	Encyclopedia	67	Chapters	927
Reviews	79	Review articles	837	Reference work entry	138
Systematic Reviews	3	Conference abstracts	38	Conference papers	69
Randomized controlled trials	7	-	-	Protocol	16
Clinical Trials	9	-	-	-	-
Meta-analysis	1	-	-	-	-

**Table 2:** Types and numbers of published studies on fibroin in online libraries.

Libraries	PubMed		Science Direct		SpringerLink
Articles	6001	Research articles	7495	Articles	4783
Full Texts	5252	Book chapters	2325	Research articles	3210
Associated Data	1558	Encyclopedia	228	Chapters	2650
Reviews	415	Review articles	3562	Reference work entry	382
Systematic Reviews	10	Conference abstracts	248	Conference papers	115
Randomized controlled trials	4	-	-	Protocol	51
Clinical Trials	5	-	-	-	-
Meta-analysis	1	-	-	-	-

**Table 5:** Different sericin extraction methods.

Degumming Method	Approach	Advantages	Disadvantages
Conventional	Soaps and Alkalis	Effective.	Sericin is highly degraded. Recovery is difficult. Time-consuming and high cost. It is not environmentally friendly. Effluent problems.
Chemical	Alkaline solutions	Application on a large scale. Low cost. High yield.	Sericin is highly degraded. Recovery is difficult. Purification steps are needed. It is not environmentally friendly. Effluent problems.
	Acidic solutions	Lower degree of degradation than alkali degumming.	Sericin is degraded. Purification steps are needed. It is not environmentally friendly. Effluent problems.
	Urea (with or without mercaptoethanol)	Low degree of degradation. Effective.	Purification steps are needed. Toxic to cells.
Biological	Proteolytic enzymes	Effective. Complete removal of SS. Environmentally friendly/no effluent problems	High cost. Sericin is degraded. Time-consuming.
Thermal	Boiling	Simple.	Sericin is degraded. Time-consuming.
	HTHP (autoclave)	No purification process is required. Low cost. Low toxicity. Suitable for safety evaluation. Environmentally friendly/no effluent problems	Heat-caused degradation.
Modern	Infrared	High yield and quality of silk sericin.	Extra equipment needed.
	Microwave	Short extraction time. Lower energy consumption. Environmentally friendly.	Commercial viability is limited.
	Ultrasound	Increased efficiency at lower temperatures. Environmentally friendly.	Higher equipment costs. Need for optimization.
	Steam treatment	Effective. Lower water consumption. Lower processing cost. Environmentally friendly.	Lack of selectivity. The developed process may not be suitable for industrial-scale mode operation.
	CO_2_ supercritical fluid	Improves efficiency, water, and energy conservation. Less wastewater generation, and low energy consumption.	Introduce chemicals, not well-established in the industry.
